# Supplementary material for: Facile and Green Fabrication of Carrageenan-Silver Nanoparticles for Colorimetric Determination of Cu2+ and S2−
Source: Nanomaterials (Basel). 2020 Jan 1;10(1):83. doi: 10.3390/nano10010083 (PMC7023203; doi:10.3390/nano10010083)
Supplement: Supplementary file 1 [file nanomaterials-10-00083-s001.pdf]

Supplementary Material

Facile and Green Fabrication of Carrageenan-Silver Nanoparticles for Colorimetric Determination of Cu^{2+} and S^{2-}

Yesheng Wang [1], Xueyi Dong [1], Li Zhao [1], Yun Xue [1], Xihui Zhao [1,2,*], Qun Li [1] and Yanzhi Xia [2]

[1] School of Chemistry and Chemical Engineering, Qingdao University, Qingdao 266071, China; wynnrs@163.com (Y.W.); 18764216018@163.com (X.D.); 18363996863@163.com (L.Z.); xueyun@qdu.edu.cn (Y.X.); Qunli501@163.com (Q.L.)

[2] State Key Laboratory of Bio-Fibers and Eco-Textiles, Shandong Collaborative Innovation Center of Marine Biobased Fibers and Ecological Textiles, Institute of Marine Biobased Materials, Qingdao University, Qingdao 266071, China; xyz@qdu.edu.cn

* Correspondence: zhaoxihui@qdu.edu.cn

Received: 12 December 2019; Accepted: 27 December 2019; Published: date

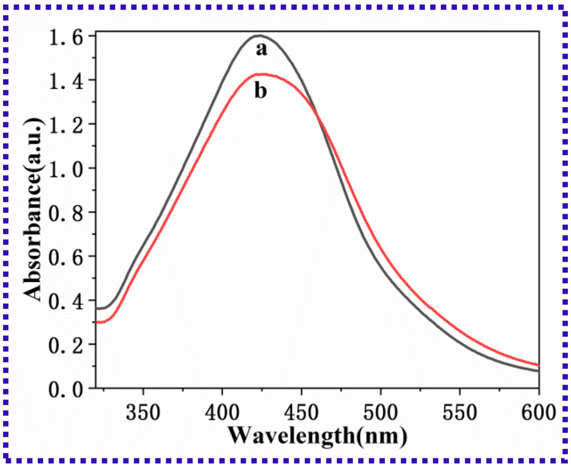

Figure S1. UV–vis spectra of carrageenan-AgNPs after reaction termination (a) and after 6 months storage at room temperature (b).

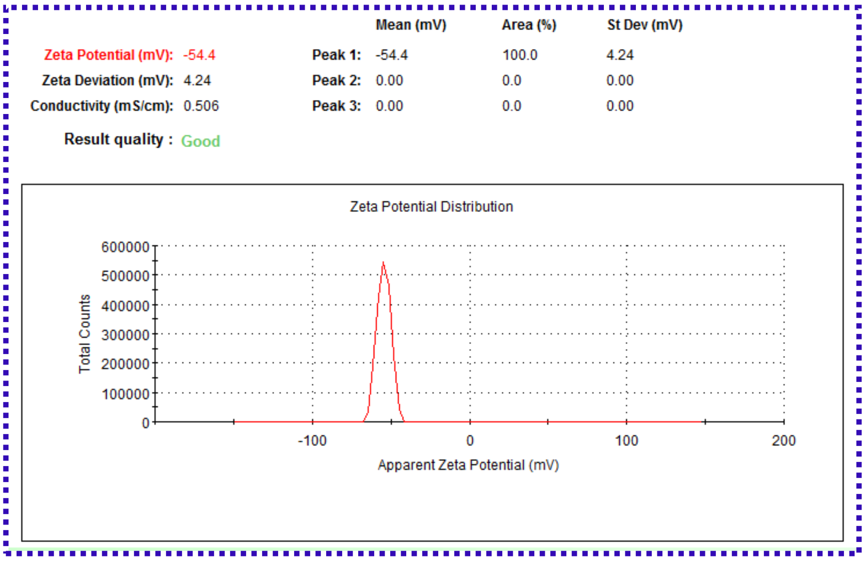

Figure S2. Zeta potential analysis of carrageenan-AgNPs prepared with 1 mM Ag^+.

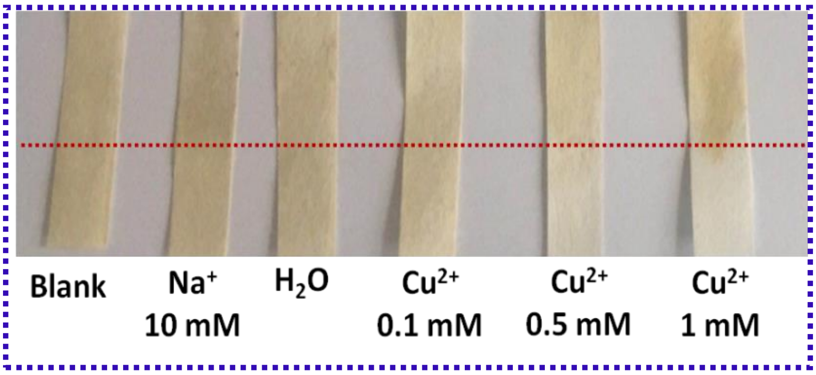

Figure S3. Photographs of test strips of carrageenan-AgNPs to various concentration of Cu^{2+}. (The lower half of the test strips were dipped into different solution.)

Table S1 Comparison of the proposed Cu^{2+} detection method with other reported methods

Methods	Probe	Linear range/(μM)	LOD/(μM)	Ref.
Fluorescent and colorimetric	F-hPEI Au NPs	0.15–23	0.077	[1]
Fluorescence	CDs	0–200	40	[2]
SWASV	AuNPs/CNFs	0.1–1.0	0.1	[3]
Colorimetric	CQ	0-18	0.53	[4]
Colorimetric	AuNPs	0.05–1.85	0.03	[5]
Colorimetric	probe S4	—	14.9	[6]
Colorimetric	R/AgNPs	0.005–0.1	0.001	[7]
Colorimetric	AgNPs	2.5–1000	1.7	This work

Table S2 Comparison of the proposed S^{2-} detection method with other reported methods

Methods	Probe	Linear range/(μM)	LOD/(μM)	Ref.
Colorimetric	Chit-AgNPs	0.8-6.4	0.35	[8]
Fluorescence	Papain-AuNCs	0.5–80.0	0.38	[9]
Colorimetric	CuNPs	12.5-50	8.1	[10]
Colorimetric	GSH-AuNPs	5.0-15	3.0	[11]
Fluorescence	Yeast-CuNCs	0–10	0.01	[12]
Electro chemistry	Au electrode	0.5–12.7	0.3	[13]
Fluorescence	sensor	0-14	0.053	[14]
Colorimetric	AgNPs	2.5–1000	2.0	This work

Table S3. Determination of Cu^{2+} and S^{2-} in tap water and lake water samples.

Samples		Concerntration of Cu^{2+} (μM)		Recovery (%)	RSD (n=3, %)
		Spiked	Measured		
tap water	1	5	5.22	104.4	5.65
	2	25	25.85	103.4	4.38
	3	50	49.75	99.5	3.97
lake water	1	5	5.4	108	3.8
	2	25	25.78	103.12	4.15
	3	50	50.45	100.9	4.45
Samples		Concerntration of S^{2-} (μM)		Recovery (%)	RSD (n=3, %)
		Spiked	Measured		
tap water	1	5	5.78	115.60	5.45
	2	25	26.50	106.00	5.22
	3	50	50.25	100.50	4.14
lake water	1	5	5.65	113.00	3.79
	2	25	24.86	99.44	3.25
	3	50	51.10	102.20	4.15

1. Wang, N.; Liu, Y.; Li, Y.; Liu, Q.; Xie, M. Fluorescent and colorimetric sensor for Cu^{2+} ion based on formaldehyde modified hyperbranched polyethylenimine capped gold nanoparticles. *Sensors Actuators, B Chem.* **2018**, *255*, 78–86.
2. Wang, L.; Li, M.; Li, W.; Han, Y.; Liu, Y.; Li, Z.; Zhang, B.; Pan, D. Rationally Designed Efficient Dual-Mode Colorimetric/Fluorescence Sensor Based on Carbon Dots for Detection of pH and Cu^{2+} Ions. *ACS Sustain. Chem. Eng.* **2018**, *6*, 12668–12674.
3. Zhang, B.; Chen, J.; Zhu, H.; Yang, T.; Zou, M.; Zhang, M.; Du, M. Facile and green fabrication of size-controlled AuNPs/CNFs hybrids for the highly sensitive simultaneous detection of heavy metal ions. *Electrochim. Acta* **2016**, *196*, 422–430.
4. Wu, C.; Wang, J.; Shen, J.; Zhang, C.; Wu, Z.; Zhou, H. A colorimetric quinoline-based chemosensor for sequential detection of copper ion and cyanide anions. *Tetrahedron* **2017**, *73*, 5715–5719.
5. Hormozi-Nezhad, M.R.; Abbasi-Moayed, S. A sensitive and selective colorimetric method for detection of copper ions based on anti-aggregation of unmodified gold nanoparticles. *Talanta* **2014**, *129*, 227–232.
6. Mohammadi, A.; Yaghoubi, S. Development of a highly selective and colorimetric probe for simultaneous detection of $Cu^{2+}$and CN^{-}based on an azo chromophore. *Sensors Actuators, B Chem.* **2017**, *251*, 264–271.
7. Basiri, S.; Mehdinia, A.; Jabbari, A. Biologically green synthesized silver nanoparticles as a facile and rapid label-free colorimetric probe for determination of Cu^{2+} in water samples. *Spectrochim. Acta - Part A Mol. Biomol. Spectrosc.* **2017**, *171*, 297–304.
8. Shanmugaraj, K.; Ilanchelian, M. Colorimetric determination of sulfide using

chitosan-capped silver nanoparticles. *Microchim. Acta* **2016**, *183*, 1721–1728.
9. Wang, L.; Chen, G.; Zeng, G.; Liang, J.; Dong, H.; Yan, M.; Li, Z.; Guo, Z.; Peng, L. Fluorescent sensing of sulfide ions based on papain-directed gold nanoclusters. **2015**, 9306–9312.
10. Hatamie, A.; Zargar, B.; Jalali, A. Talanta Copper nanoparticles : A new colorimetric probe for quick , naked-eye detection of sul fi de ions in water samples. *Talanta* **2014**, *121*, 234–238.
11. Links, D.A. Highly specific colorimetric recognition and sensing of sulfide with glutathione-modified gold nanoparticle probe based on an anion-for-molecule ligand exchange reaction †. **2012**, 1556–1558.
12. Jin, L.; Zhang, Z.; Tang, A.; Li, C.; Shen, Y. Biosensors and Bioelectronics Synthesis of yeast extract-stabilized Cu nanoclusters for sensitive fl uorescent detection of sul fi de ions in water. *Biosens. Bioelectron.* **2016**, *79*, 108–113.
13. Yang, Y.; Yang, M.; Wang, H.; Jiang, J.; Shen, G.; Yu, R. An amperometric horseradish peroxidase inhibition biosensor based on a cysteamine self-assembled monolayer for the determination of sulfides. **2004**, *102*, 162–168.
14. Cheng, F.; Wu, X.; Lon, Y.; Chen, G.; Zeng, R. Sensors and Actuators B : Chemical A porphyrin-based near-infrared fluorescent sensor for sulfur ion detection and its application in living cells. *Sensors Actuators B. Chem.* **2016**, *228*, 673–678.
